# Peripheral blood and tumor biomarkers in patients with advanced melanoma treated with combination nivolumab (anti-PD-1, BMS-936558, ONO-4538) and ipilimumab

**DOI:** 10.1186/2051-1426-1-S1-O6

**Published:** 2013-11-07

**Authors:** Margaret K Callahan, Christine E Horak, Michael A Curran, Travis Hollman, David A Schaer, Jianda Yuan, Alexander M Lesokhin, Shigehisa Kitano, Quan Hong, Charlotte E Ariyan, Klaus J Busam, William Feely, Maria Jure-Kunkel, Joseph Grosso, Jason S Simon, Alan J Korman, Jon M Wigginton, Ashok K Gupta, Xiaoling Zhang, Therese Phillips, Pauline Simmons, Mario Sznol, Jedd D Wolchok

**Affiliations:** 1 Memorial Sloan-Kettering Cancer Center, New York, NY, USA; 2 Bristol-Myers Squibb, Princeton, NJ, USA; 3 Bristol-Myers Squibb, Redwood City, CA, USA; 4 Dako North America, Inc., Carpinteria, CA, USA; 5 Yale University, New Haven, CT, USA

## Background

Programmed death-1 (PD-1) and cytotoxic T-lymphocyte antigen-4 (CTLA-4) receptors are immune checkpoint receptors involved in tumor immune evasion. Nivolumab and ipilimumab are fully human monoclonal PD-1 and CTLA-4 blocking antibodies, respectively, and have shown clinical antitumor activity as monotherapy. In a multicohort, phase 1 study with melanoma patients (pts), nivolumab/ipilimumab combination produced objective response rates (ORRs) of up to 53% (NCT01024231). We evaluated putative predictive biomarkers for nivolumab and ipilimumab monotherapy in the combination setting, including tumor PD-L1 expression, absolute lymphocyte count (ALC), peripheral blood (PB) myeloid-derived suppressor cells (MDSCs), and pharmacodynamic changes in T-cell phenotype.

## Methods

An automated immunohistochemistry assay (developed by Dako) using the 28-8 PD-L1 antibody was used to evaluate tumor PD-L1 membrane expression in archival FFPE specimens. ALC was measured in serial PB samples. Flow cytometry was used to characterize changes in percentage, number, and phenotype of activated CD4^+^ and CD8^+^ T cells and MDSC.

## Results

Using a cut-off of 5% tumor cell membrane staining, 37% of pts (13/35) exhibited PD-L1 expression. Nine of 22 pts (41%) with PD-L1 negative tumors and 6/13 pts (46%) with PD-L1 positive tumors achieved an OR. ALC increase vs baseline was not observed, but phenotypic changes in PB T-cell subsets (increases in percentage of CD4 and CD8 expressing HLA-DR, ICOS, and/or Ki67) were evident. Six of 14 pts (43%) with low ALC (<1.0 at wk 6-7) achieved an OR, vs 15/38 pts (39%) with ALC ≥1.0 at wk 6-7. Lower pretreatment percent MDSC correlated with higher ORR (*P*<0.05).

## Conclusions

ORRs with nivolumab/ipilimumab combination in this small pt subset were not associated with PD-L1 expression or ALC, in contrast to prior observations with nivolumab or ipilimumab monotherapy, respectively. Compared with either therapy alone, the combination may induce an immune response with distinct characteristics. PB and tumor biomarkers, and their relationship to clinical activity patterns, will be further investigated in this study and in phase 2/3 randomized studies.

